# A case of Epstein-Barr virus–associated hemophagocytic lymphohistiocytosis with severe cardiac complications

**DOI:** 10.1186/s12887-016-0718-3

**Published:** 2016-10-28

**Authors:** Yoshiki Kawamura, Hiroki Miura, Yuji Matsumoto, Hidetoshi Uchida, Kazuko Kudo, Tadayoshi Hata, Yoshinori Ito, Hiroshi Kimura, Tetsushi Yoshikawa

**Affiliations:** 1 Department of Pediatrics, Fujita Health University School of Medicine, 1-98 Dengakugakubo, Kutsukake-cho, Toyoake, 470-1192 Japan; 2 Faculty of Medical Technology, School of Health Science, Fujita Health University, 1-98 Dengakugakubo, Kutsukake-cho, Toyoake, 470-1192 Japan; 3 Department of Pediatrics, Nagoya University Graduate School of Medicine, 65 Tsurumai-cho, Showa-ku, Nagoya, 466-8550 Japan; 4 Department of Virology, Nagoya University Graduate School of Medicine, 65 Tsurumai-cho, Showa-ku, Nagoya, 466-8550 Japan; 5 Present address: Division of Viral Products, Office of Vaccines Research and Review, Center for Biologics Evaluation and Research, Food and Drug Administration, 10903 New Hampshire Avenue, Silver Spring, MD 20993 USA

**Keywords:** Epstein-Barr virus, Hemophagocytic lymphohistiocytosis, Coronary artery lesion, Myocarditis, Rituximab

## Abstract

**Background:**

Hemophagocytic lymphohistiocytosis (HLH) is a life threatening hematological disorder associated with severe systemic inflammation caused by an uncontrolled and ineffective immune response resulting in cytokine storm. Epstein-Barr virus (EBV) is the most common infectious agent in patients with the viral-associated HLH. Limited numbers of cases with cardiac complication have been demonstrated in other viral-associated HLH patients. Herein, we report a pediatric case of severe EBV-associated HLH with cardiac complications.

**Case presentation:**

A previously healthy 4-year-old Japanese female was admitted to a local hospital with a four day history of fever. Despite antibiotic treatment, her fever persisted to day 7 of the illness. Finally, the diagnosis of HLH was confirmed by fulfilling diagnostic criteria for HLH and pathological analysis of bone marrow aspiration. Real-time PCR detected a high copy number of EBV DNA in the peripheral blood mononuclear cells (PBMCs) at the time of hospital admission. During treatment according to HLH-2004 protocol, sudden cardiopulmonary arrest (CPA) occurred on day 30 of the illness and immediate resuscitation was successful. Acute myocarditis was considered the cause of the CPA. Although the treatment regimen was completed on day 88 of the illness, a remarkably high copy number of EBV DNA was still detected in her PBMCs. Based on our flow cytometric in situ hybridization analysis that revealed EBV infection of only B lymphocytes, we decided to administer rituximab to control the abnormal EBV infection. Afterwards the amount of EBV DNA decreased gradually to undetectable level on day 130 of the illness. Unfortunately, a coronary artery aneurysm was discovered at the left main coronary artery on day 180 of the illness. Finally, the patient was discharged from the hospital on day 203 of the illness without sequelae except for a coronary aneurysm.

**Conclusions:**

In this case report, EBV-HLH was complicated with cardiac symptoms such as myocarditis and coronary artery aneurysm. Although remarkably high copy number of EBV DNA was detected in PBMCs after completion of the HLH-2004 protocol, rituximab treatment resulted in a dramatic decrease of EBV DNA to undetectable levels. Rituximab treatment might have been beneficial for the patient’s survival.

## Background

Hemophagocytic lymphohistiocytosis (HLH) is a life threatening hematological disorder associated with severe systemic inflammation caused by an uncontrolled and ineffective immune response, such as activation and aberrant proliferation of macrophages, lymphocytes, and dendritic cells resulting in cytokine storm [1]. HLH is divided into two types based on its etiology: primary (genetic) and secondary (acquired) HLH. Primary HLH is due to genetic defects in cellular cytotoxicity, whereas secondary HLH is associated with viral infections including Epstein-Barr virus (EBV), autoimmune diseases, malignant diseases, and acquired immune deficiency conditions. EBV is the most common infectious agent in patients with the viral-associated HLH.

Limited numbers of cases with cardiac complication have been demonstrated in the viral-associated HLH patients [2, 3]. Additionally, distinguishing between HLH and Kawasaki disease (KD) that also causes hypercytokinemia was difficult because of similar clinical symptoms [4]. KD is one of the most common vasculitis of childhood which shows characteristic bilateral nonexudative conjunctivitis, erythema of the lips and oral mucosa, rash, extremity changes, and cervical lymphadenopathy. It is well known that KD can cause arteritis resulting in coronary aneurism. Coronary artery aneurism was observed in the patients with HLH, who fulfilled the diagnostic criteria of KD [5].

In addition to HLH, in some rare cases EBV can cause a chronic active EBV (CAEBV) infection, which is a non-familial syndrome that reflects a specific immunodeficiency and impairment of host responses against EBV [6]. In immunocompetent individuals, EBV establishes a latent infection in B lymphocytes after primary viral infection. However, in some cases of EBV infection a chronic active viral infection of T lymphocytes and natural killer cells may be detected. Previous studies have described CAEBV patients with cardiac complications, including coronary artery aneurism [7]. Thus, not only HLH but also CAEBV can cause severe cardiac symptoms similar to Kawasaki disease. Herein, we report a pediatric case of severe EBV-associated HLH that did not fulfill with criteria for KD with cardiac complications including myocarditis and a coronary artery aneurism.

## Case presentation

**Fig. 1:**
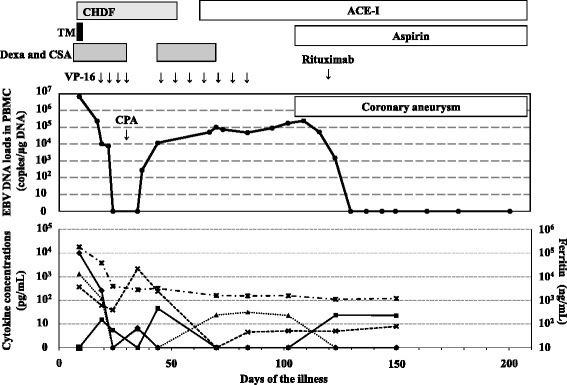
Clinical course of the patient and kinetics of EBV-DNA load and biomarkers. The patient had a rapid decrease in EBV-DNA load after initial treatments with chemotherapy that induced severe bone marrow suppression. No mononuclear cells were obtained from the patient during this period. CHDF**,** continuous hemodiafiltration; ACE-I, angiotensin-converting enzyme inhibitor; TM, thrombomodulin alpha; Dexa, dexamethasone; CSA, cyclosporine; VP-16, etoposide; CPA, cardiopulmonary arrest; EBV, Epstein-Barr virus, IFN; interferon, IL; interleukin, TNF; tumor necrosis factor

A previously healthy 4-year-old Japanese female was admitted to a local hospital with a four day history of fever as high as 39 °C. Although she had pharyngitis, lymphoadenopathy and hepatosplenomegaly, no symptom suggesting KD such as conjunctivitis, extremity changes and skin rash was demonstrated at the time of admission. Despite antibiotic treatment, her fever persisted to day 7 of the illness. HLH was suspected because of pancytopenia (WBC, 1000/μL [4000-9000/μL]; Plt, 2.7 × 10^4^/μL [20-40 × 10^4^/μL]; Hb, 4.2 g/dL [12-15 g/dL]) as well as elevated levels of ferritin (greater than 40000 ng/mL [24-336 ng/mL]) and LDH (5540 IU/L [140-180 IU/L]). Finally, the diagnosis of HLH was confirmed by fulfilling diagnostic criteria for HLH and pathological analysis that revealed hemophagocytosis in a bone marrow sample [1]. She was transferred to our hospital on day 9 of the illness because she failed to respond to the conventional treatments for HLH, including dexamethasone, cyclosporine, and a high dose of immunoglobulins. The patient’s treatment course and cytokine profile is summarized in Fig. 1.

At the time of admission to our hospital, she was unconscious (Glasgow coma scale was E2V2M4) with disseminated intravascular coagulation (DIC) and acute renal injury (blood urea nitrogen, 22.5 mg/dL; creatinine, 0.69 mg/dL). Therefore, in addition to recombinant thrombomodulin that has been suggested to be effective treatment for DIC patients [8], continuous hemodiafiltration was also carried out. Real-time PCR detected a high copy number of EBV DNA (6.6 × 10^6^ copies/μg DNA) in the peripheral blood mononuclear cells (PBMCs) at the time of hospital admission. Although EBV-VCA-IgM, -IgA, EBV-EA DR-IgG, -IgA, and EBV-EBNA IgG titers were negative, EBV-VCA-IgG was positive (×80) by immunofluorescence assay. Additionally, EBV-infected cells were identified as T and B lymphocytes using the flow cytometric in situ hybridization method [9]. Again, this patient did not fulfill the diagnostic criteria of Kawasaki disease because of lacking conjunctivitis, extremity changes and skin rash during the observation period [10].

Despite intensive treatments, her condition deteriorated, and etoposide (VP-16) administration was started on day 19 of the illness according to the treatment guideline, HLH-2004 [1]. After starting the chemotherapy, DIC gradually improved and the EBV-DNA load decreased. However, sudden cardiopulmonary arrest (CPA) occurred on day 30 of the illness and immediate resuscitation was successful. Acute myocarditis was considered the cause of the CPA because elevated N-terminal pro-brain natriuretic peptide (49554 pg/mL [<300 pg/mL] ) and cardiac markers (creatine kinase (CK), 189 IU/L [22-198 IU/L] ; CK-myoglobin, 12.4 ng/mL [<4.3 ng/mL] ; troponin I, 0.40 ng/mL [<0.03 ng/mL] ), an enlargement of the cardiac silhouette in chest X-ray, low QRS voltage at the chest lead electrocardiogram, and abnormal echocardiography findings such as myocardial thickness were observed. Angiotensin-converting enzyme inhibitor was started for myocardial protection, and dexamethasone, cyclosporine, and VP-16 administrations were discontinued from days 30 to 46 of the illness.

**Fig. 2:**
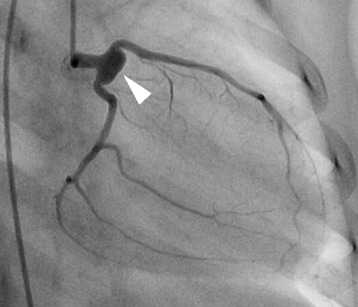
Left coronary angiography on the day 180 of the illness. Injection of the left coronary artery demonstrated a large, saccular aneurysm in the proximal left anterior descending and circumflex coronary arteries (*White arrow*)

Although the treatment regimen based on the HLH-2004 protocol was completed on day 88 of the illness and the general condition of the patient was good, a remarkably high copy number of EBV DNA was still detected in her PBMCs (50,193–241,284 copies/μg DNA). Based on our flow cytometric in situ hybridization analysis that revealed EBV infection of only B lymphocytes, we decided to administer rituximab (375 mg/m^2^) only one time to control the abnormal EBV infection. The amount of EBV DNA decreased gradually to undetectable level on day 130 of the illness. Although lymphocytopenia (Minimum lymphocyte 0/μL) were observed for a month after administration of rituximab, it spontaneously improved. Unfortunately, a coronary artery aneurysm was discovered at the left main coronary artery and aspirin was administered from day 105 of the illness. Subsequent coronary angiography showed an 8 × 4 mm sized coronary artery aneurysm at the left coronary artery on day 180 of the illness (Fig. 2). The patient was eventually discharged from the hospital on day 203 of the illness without sequelae except for a coronary artery aneurysm on day 203 of the illness.

In order to elucidate pathophysiology of the patient, kinetics of serum ferritin and levels of cytokines including interferon (IFN)-γ, tumor necrosis factor (TNF)-α, interleukin (IL) -2, 4, 6, and 10 were examined (Fig. 1). The concentrations of cytokines were measured by the cytometric bead array kit–BD^TM^ CBA Human Th1/Th2 Cytokine Kit (BD Biosciences, San Jose, CA). Although serum ferritin, IFN-γ (9993 pg/mL), IL-6 (368 pg/mL) and IL-10 (1322 pg/mL) concentrations were elevated remarkably at the time of hospital admission, IFN-γ and IL-10 levels decreased quickly and ferittin and IL-6 also decreased slightly after starting the intensive treatments. Transient increases in levels of IL-6, IFN-γ, TNF-α and IL-10 concentrations corresponding to an increase in EBV DNA load were demonstrated probably due to the recovery of bone marrow.

## Conclusions

Both HLH and CAEBV infection can cause hypercytokinemia, which may be associated with cardiac complications such as myocarditis and coronary artery aneurysms [6]. Although several etiologies have been suggested to be involved in HLH, to date only one EBV-HLH case with a coronary artery aneurysm has been reported [5]. The previously reported EBV-HLH case fulfilled the diagnostic criteria of Kawasaki disease and was initially treated with high-dose intravenous immunoglobulins and oral aspirin. The patient in that report was subsequently diagnosed with EBV-HLH because of prolonged hepatosplenomegaly, abnormal laboratory data suggesting hypercytokinemia, and the pathological finding of hemophagocytosis. Meanwhile, our case lacked the typical symptoms suggestive of Kawasaki disease such as conjunctivitis, extremity changes and skin rash. Additionally, primary EBV infection that is one of the most important infectious diseases for ruling out for diagnosis of Kawasaki disease was demonstrated in soon after admission to the previous hospital. Thus, although these two cases are both considered to be EBV-HLH, the previously reported case and our current case had distinct clinical courses of disease. Thus, in this case report EBV-HLH lacking clinical symptoms of Kawasaki disease was associated with cardiac complications such as myocarditis and coronary artery aneurysm. Parvovirus and Enterovirus have been suggested to be involved in infection-associated HLH complicating myocarditis [2, 3]. This case report supports the notion that EBV can cause not only HLH but also severe cardiac complications such as myocarditis and coronary artery aneurysm.

It is possible that lacking of genetic analysis such as mutations in the gene encoding perforin or in X-linked lymphoproliferative disease might be limitation of this study. However, EBV infection was confirmed at the onset of HLH, and the parents didn’t have a consanguineous marriage. Moreover, this case was female and didn’t have family medical history. Therefore it was little chance to be diagnosed with primary HLH.

Kinetics of the cytokines in our patient was consistent with a previous study which analyzed 24 children with HLH, which demonstrated the significant increase of IFN-γ (median level; 901.7 pg/mL), IL-6 (median level; 63.8 pg/mL) and IL-10 (median level; 879.0 pg/mL) in the acute phase [11]. The same method was used in the previous study and the present case analysis to measure concentrations of the cytokines. In comparison to the study, the levels of the 3 cytokines (IFN-γ; 9993 pg/mL, IL-6; 368 pg/mL, and IL-10; 1322 pg/mL) were remarkably high in acute phase sera obtained from our patient. Thus, remarkably high levels of the cytokines may play an important role in causing severe clinical manifestations including coronary artery aneurysm in this patient. However, as serum cytokine concentrations decreased temporarily at the time of CPA, preceding high levels of cytokines may lead to severe cardiac damage in the patient.

Unfortunately, the management of EBV-HLH remains challenging. Despite the various intensive treatments, including bone marrow transplantation, more than 30 % of the patients diagnosed with EBV-HLH succumb to the disease or its complications [12]. B-cell depleting agents are not always an effective treatment strategy in HLH patients because EBV can infect either B lymphocytes or non-B lymphocytes. In a previous report, only 43 % of HLH patients showed improvements following the administration of B-cell depleting agents; however, an initial course of a rituximab-containing regimen may rapidly reduce EBV-DNA load [13]. In our case, rituximab was not administered initially, because the initial flow-cytometric in situ hybridization analysis identified not only B lymphocytes but also T lymphocytes as the EBV-infected cells. However, rituximab was eventually administered once we identified only EBV-infected B lymphocytes. It has been demonstrated that cell fractions of EBV infected cells may change during the observation period in the HLH patient [14]. Although the general condition of the patient was not so severe after completion of the HLH-2004 protocol, a remarkably high copy number of EBV DNA was detected in PBMCs. Rituximab treatment resulted in a dramatic decrease of EBV DNA to undetectable levels. Interestingly, previous reports suggested that the rapid decrease of EBV-DNA load following rituximab treatment was beneficial for the patient’s survival [15]. Therefore, it is expected that the prognosis for our patient is good despite her cardiac sequelae.

## Abbreviations

CAEBV: Chronic active Epstein-Barr virus
CK: Creatine kinase
CPA: Cardiopulmonary arrest
DIC: Disseminated intravascular coagulation
EBV: Epstein-Barr virus
HLH: Hemophagocytic lymphohistiocytosis
IFN: Interferon
IL: Interleukin
PBMCs: Peripheral blood mononuclear cells
TNF: tumor necrosis factor
VP-16: etoposide
